# Assessment of rotational dose perturbations in SBRT: A radiomic‐dosiomic predictive model based on the structural similarity index measure

**DOI:** 10.1002/acm2.70634

**Published:** 2026-05-26

**Authors:** Rui Qi, Jinglin Sun, Tingyu Mu, Hongji Shi, Jiayi Zhang, Xiangkun Dai

**Affiliations:** ^1^ Department of Radiation Oncology The First Medical Center of PLA General Hospital Beijing China

**Keywords:** dose distribution consistency, radiomics, dosiomics, rotational setup error, SBRT

## Abstract

**Background:**

Stereotactic body radiation therapy (SBRT) delivers ablative doses with high precision but is sensitive to target motion, especially rotational errors. Conventional dose metrics (dose difference (DD), distance‐to‐agreement (DTA), gamma index) show limited sensitivity. The structural similarity index measure (SSIM), combined with radiomics and dosiomics, may better characterize complex dose perturbations.

**Purpose:**

To evaluate the impact of rotational errors on SBRT dose distributions using SSIM and to develop predictive models integrating radiomic‐dosiomic features.

**Materials and methods:**

Sixty‐nine clinically validated CyberKnife treatment plans were retrospectively analyzed. In a two‐stage phantom experiment, dose distributions were measured under simulated discrete and non‐discrete rotational errors (ranging from 1.5 to 4.5 degrees), verified by the CyberKnife System Plan QA module. Of the total dose images acquired, 405 datasets (364 from the first stage and 41 from the second stage) passed rigorous quality control. SSIM and gamma passing rates (at 1%/1 mm,1.5%/1.5 mm and 2%/2 mm criteria) were calculated to evaluate their relative sensitivity. Radiomic and dosiomic features were extracted, followed by feature selection using eXtreme Gradient Boosting (XGBoost) with Shapley Additive Explanations (SHAP). Predictive models were optimized via automated machine learning. Performance was assessed with coefficient of determination (R^2^), mean absolute error (MAE), root mean ‐squared error (RMSE), and median absolute error (MedAE).

**Results:**

Rotational errors significantly decreased SSIM, showing robust negative correlations with rotation angles across all single‐directional inputs (r ranging from −0.632 to −0.747, all *p* < 0.001). Notably, in multi‐directional scenarios where conventional gamma metrics lost statistical significance (*p* > 0.05), SSIM retained a statistically significant negative correlation (*p* < 0.001), demonstrating superior sensitivity. The radiomic‐dosiomic model accurately predicted SSIM (R^2^ = 0.818, MAE ≈ 0.0096, RMSE ≈ 0.0123, MedAE ≈ 0.0080). SHAP analysis highlighted wavelet‐transformed dosiomic features as key predictors, and the inclusion of multi‐stage data ensured the model's generalizability across diverse patient characteristics.

**Conclusions:**

SSIM is a sensitive, structure‐aware metric for evaluating rotational dose perturbations in SBRT, particularly surpassing conventional metrics in detecting complex multi‐directional errors. Integrating radiomic‐dosiomic modeling enhances predictive accuracy and robustness, providing a reliable tool for individualized and automated radiotherapy quality assurance (QA).

## 1 INTRODUCTION

Stereotactic body radiation therapy (SBRT) is a high‐precision external beam radiotherapy technique that delivers ablative doses to well‐defined tumor targets in only a few fractions. Owing to its steep dose gradients and limited fractionation, SBRT demands sub‐millimeter geometric accuracy to achieve optimal target coverage while sparing adjacent organs at risk (OARs).^1^ Maintaining such precision requires effective restriction or tracking of target motion during both treatment planning and delivery.

However, in clinical practice, rotational setup errors remain difficult to correct in real time. In CyberKnife treatments, rotational compensation typically requires at least three fiducial markers, yet implantation may be infeasible due to tumor size, anatomical constraints, or patient condition, and even implanted markers may migrate or fail over time.^2^, ^3^ When only translational corrections are applied, uncorrected rotational errors can lead to substantial degradation in dose–volume coverage and increased OAR dose uncertainty. Quantitatively characterizing the dosimetric consequences of rotational perturbations is therefore of critical importance for treatment quality assurance (QA) and adaptive SBRT planning.

Traditionally, planar dose evaluation relies on dose difference (DD), distance‐to‐agreement (DTA), and the combined gamma index. While DD is overly sensitive in high‐gradient regions, DTA tends to under‐respond, and the gamma index—although widely adopted—often exhibits weak correlation with clinically meaningful dose discrepancies.^4^, ^5^, ^6^ Moreover, the gamma framework neglects spatial structural information, making it difficult to localize or interpret the origin of distribution discrepancies.^7^ Consequently, there is growing interest in metrics that incorporate structural or perceptual similarity to better reflect the spatial coherence of dose deviations.

The structural similarity index measure (SSIM), originally proposed for image quality assessment,^8^ compares two images by evaluating luminance, contrast, and structural fidelity. When applied to radiotherapy dose distributions, SSIM offers enhanced sensitivity to structural perturbations compared with conventional scalar metrics and has shown promise for QA and delivery error detection.^9^, ^10^ Recent work has further demonstrated its applicability to three‐dimensional patient‐specific QA and its sensitivity to delivery errors in high‐gradient dose fields.^11^ Yet, prior studies have largely treated SSIM as a global similarity score, without exploring its directional sensitivity or underlying spatial determinants when rotational errors occur in SBRT dose fields.

Parallel to these developments, radiomics and dosiomics have emerged as data‐driven approaches for quantifying spatial heterogeneity in medical images and dose distributions. By extracting high‐dimensional texture and statistical features, these methods capture subtle geometric and intensity patterns associated with treatment complexity, delivery accuracy, and biological response.^11^, ^12^, ^13^, ^14^ Recent studies have demonstrated that dosiomic features can predict gamma passing rates^12^ or correlate with post‐treatment biochemical outcomes,^13^ suggesting that dose‐texture characteristics may encode intrinsic sensitivity to setup perturbations.

Building on these insights, the present study investigates the direction‐dependent structural degradation of SBRT dose distributions induced by rotational setup errors, integrating SSIM with radiomic–dosiomic feature modeling. We hypothesize that rotational perturbations introduce anisotropic distortions in dose topology, which can be quantitatively characterized by SSIM and explained through multiscale spatial features extracted from the dose field.^15^ Using phantom‐based experiments with controlled rotational deviations, we evaluate the sensitivity and robustness of SSIM relative to conventional metrics, identify key dosiomic determinants of structural degradation, and construct predictive models linking geometric errors to structural dose integrity. This integrative framework aims to advance the scientific understanding of rotational error effects and to support data‐driven, individualized QA strategies for SBRT.

## 2 MATERIALS AND METHODS

### 2.1 Dosimetric measurement and analysis

**FIGURE 1:**
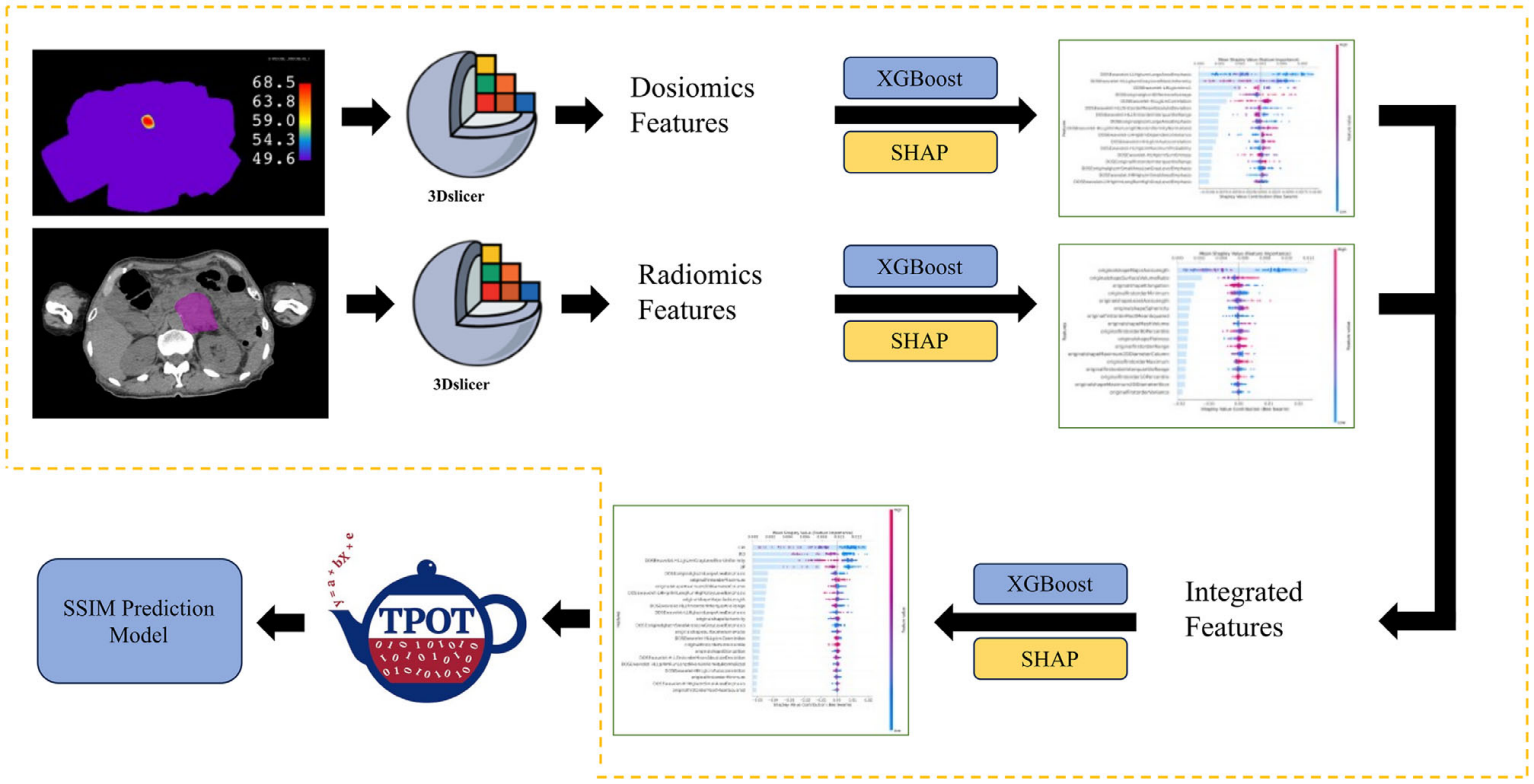
Schematic diagram of the study workflow.

To acquire two‐dimensional dose distributions under different rotational error conditions and evaluate their SSIM against the reference plan, we employed the SRS MapCHECK detector system (Sun Nuclear Corporation, USA) in combination with the StereoPHAN phantom. The SRS MapCHECK system is equipped with 1,013 highly sensitive semiconductor detectors, each with a volume of 0.007 mm^3^ (0.48 mm × 0.48 mm × 0.03 mm), providing excellent spatial resolution suitable for high‐precision two‐dimensional dose verification of stereotactic radiotherapy (SRT) plans. The overall study workflow is shown in Figure 1.

Measurements were performed using the SRS MapCHECK integrated with a cylindrical StereoPHAN phantom made of polymethyl methacrylate (PMMA). To simulate clinical buildup and backscatter conditions for more accurate dose distribution reconstruction, 2.2 cm PMMA plates were positioned both above and below the detector. To model potential clinical rotational errors, taking the mechanical isocenter (also the plan isocenter point) defined by the CyberKnife treatment planning system (TPS) as the axis, rotation errors were simulated by adjusting the posture of the StereoPHAN phantom on the 6D couch to specific angles, each of which was verified using the CyberKnife 6D tracking module. Corresponding 2D dose distributions were then recorded by repeatedly delivering treatment plans at each specified orientation. The data acquisition process was divided into two distinct phases. In the first phase, dose distributions were collected at 15 fixed angles for each patient to investigate the specific impact of rotation on dose delivery. In the second phase, patients were grouped and assigned to designated angles to enhance the generalization of subsequent modeling, ensuring the model's robustness across diverse patient characteristics.

All acquired two‐dimensional dose data were interpolated to a 45 × 45 matrix to standardize spatial dimensions across measurements. Considering that the effective detection area of SRS MapCHECK is 77.00 mm × 77.00 mm and that clinical evaluation primarily focuses on the high‐dose region (≥ 70% of the maximum dose), dose values below the 70% maximum dose threshold were set to zero to eliminate low‐dose interference. These preprocessing steps were implemented in Python to ensure automation and consistency. In addition, to bridge the gap between image‐based similarity and clinical relevance, and considering the lengthy time required for the TPS to calculate three‐dimensional dose distributions, we selected a subset of samples for a simple preliminary analysis and calculated a representative DVH‐based metric, ΔV100. This metric was defined as the difference in target V100 between the non‐rotated and rotated conditions, and was used to quantify the loss of prescription‐dose target coverage caused by rotational errors. Pearson correlation analysis was then conducted to assess the relationship between SSIM and ΔV100, ensuring a direct link between structural dose integrity and treatment quality. The detailed data acquisition protocol, specifying the specific rotational error scenarios and their distribution across the cohort, is provided in Table S1.

To assess the structural effects of rotational errors on dose distribution, the two‐dimensional dose matrices at each rotation angle were compared with the corresponding error‐free (0°) reference using SSIM. SSIM evaluates image similarity based on luminance, contrast, and structural components:

$$\begin{array}{cc} \mathrm{SSIM}(x,y) & ={\left[l(x,y)\right]}^{\alpha }{\left[c(x,y)\right]}^{\beta }{\left[s(x,y)\right]}^{\gamma }\\ l(x,y) & =\frac{2\mu_{x}\mu_{y}+C_{1}}{\mu_{x}^{2}+\mu_{y}^{2}+C_{1}}\\ c(x,y) & =\frac{2\delta_{x}\delta_{y}+C_{1}}{\delta_{x}^{2}+\delta_{y}^{2}+C_{2}}\\ s(x,y) & =\frac{\delta_{\mathrm{xy}}+C_{3}}{\delta_{x}\delta_{y}+C_{3}}\\ C_{1} & ={(K_{1}L)}^{2},\;C_{2}={(K_{2}L)}^{2},\;C_{3}=C_{2}/2 \end{array}$$

where *l*(x,y) denotes luminance comparison, *c*(x,y) denotes contrast comparison, *s*(x,y) and denotes structural comparison. Here, **μ_x_** and **μ_y_** represent the means of images X and Y; **δ_x_^2^** and **δ_y_^2^** represent variances; denotes covariance; and ***C*_1_** and ***C*_2_** are constants preventing denominator instability. Parameters **α**, **β**, and **γ** weight luminance, contrast, and structure, respectively; when set to 1, the SSIM value ranges from 0 to 1, with higher values indicating greater similarity. All SSIM calculations were implemented using the scikit‐image library in Python.

$$\mathrm{SSIM}(x,y)=\frac{(2\mu_{x}\mu_{y}+C_{1})(2\delta_{\mathrm{xy}}+C_{2})}{(\mu_{x}^{2}+\mu_{y}^{2}+C_{1})(\delta_{x}^{2}+\delta_{y}^{2}+C_{2})}$$

### 2.2 Radiomics and dosiomics feature extraction

Feature extraction was performed using the PyRadiomics module integrated within the three‐dimensional (3D) Slicer platform. PyRadiomics is an open‐source, standardized Python toolkit that extracts high‐dimensional quantitative features (e.g., intensity, shape, texture) from medical images and has been widely applied in tumor phenotyping and treatment response prediction.

To improve comparability across data sources, all images underwent uniform preprocessing before feature extraction. Both imaging and dose distribution data were resampled to a consistent voxel spacing to avoid resolution‐related bias, and intensity/dose values were discretized into fixed‐width bins to stabilize texture matrix construction and reduce computational sensitivity.

For radiomics feature extraction, we focused on first‐order statistical features and three‐dimensional shape features derived directly from the original, unfiltered images, given their relative robustness to angular perturbations in radiotherapy.

For dosiomics feature extraction, we comprehensively characterized the spatial structure and intensity variation of the three‐dimensional dose field. In addition to first‐order features, we extracted texture‐based heterogeneity indices, including gray‐level co‐occurrence matrix (GLCM), gray‐level run‐length matrix (GLRLM), and gray‐level size‐zone matrix (GLSZM) features, as well as multiscale wavelet‐based features to capture both local and global distribution patterns. All features were extracted from resampled and discretized dose volumes under a standardized framework to ensure reproducibility.

Finally, imaging and dosimetric features were paired with their respective rotation angle labels to form two structured datasets for subsequent feature selection and modeling.

### 2.3 Feature engineering and model development

To prevent data leakage and ensure true generalizability, the dataset was split into training (70%) and testing (30%) cohorts using a grouped split strategy based on individual treatment plans. Specifically, all rotated scenarios derived from the same base plan were assigned exclusively to either the training or the testing set, ensuring that the model was validated on entirely unseen patient geometries. Within the training cohort, 10‐fold cross‐validation was applied to assess stability and consistency of feature selection, thereby reducing variability due to random sampling.

Prior to modeling, all features underwent normalization and selection. Numerical features were scaled to the [0,1] range using min–max normalization to eliminate unit disparities. Because of the high dimensionality, direct modeling risked overfitting, computational inefficiency, and reduced interpretability. Moreover, imbalances in radiomics and dosiomics feature numbers could bias model weighting. To mitigate this, each feature group was prefiltered independently.

Feature selection was performed using eXtreme Gradient Boosting (XGBoost) combined with Shapley Additive Explanations (SHAP). Separate XGBoost regression models were trained on radiomics and dosiomics datasets with SSIM as the prediction target. SHAP values quantified each feature's average marginal contribution to model output, enabling identification of both linear and nonlinear dependencies and interactions. Top‐ranked features from each subset were merged into an integrated feature set. A global XGBoost model was then retrained, and SHAP values were recalculated for joint feature evaluation. Only features with significant global contributions were retained, which were concatenated with rotation angle data to form the final input set for regression modeling.

Automated machine learning with TPOT^16^ was used to construct regression models based on the final feature set, with SSIM as the prediction target. TPOT applies genetic programming to optimize preprocessing pipelines, model selection, and hyperparameters, thereby minimizing manual intervention and enhancing stability. Candidate models were assessed by 10‐fold cross‐validation, with the coefficient of determination (R^2^) as the primary metric.

Additionally, input schemes incorporating single‐plane (axial, sagittal, coronal), dual‐plane, and triple‐plane (multimodal fusion) perspectives were tested to evaluate the impact of directional combinations on dosimetric assessment. Furthermore, to isolate and evaluate the independent predictive value of radiomic‐dosiomic features versus geometric error parameters, three distinct modeling scenarios were compared: one using the combined feature set (full model), one using only rotation angles, and one using only radiomic and dosiomic features with angles excluded.

### 2.4 Statistical analysis

For the statistical analysis, datasets from both acquisition phases were pooled to evaluate the correlations between dosimetric indices and angular deviations. The study was conducted in two stages, with the first stage focusing on single‐directional inputs, including upward pitch (UP), rotation (RO), and clockwise yaw (CW). Within this framework, the correlation between various dosimetric metrics and the clinical acceptability of each rotational angle was calculated. The primary evaluation criterion employed was the gamma analysis passing rate, with a threshold of > 95% under the 2%/2 mm criterion, aligning with standard clinical practice. The dosimetric indices analyzed included Relative Dose (RD), Absolute Dose (AD), DD, and SSIM. Here, “2%/2 mm” refers to the combined gamma analysis criteria. To investigate whether a specific gamma threshold could replicate SSIM's sensitivity, intermediate criteria including 1.5%/1.5 mm, along with a more stringent 1%/1 mm limit, were incorporated into the comparison. This allows for a more comprehensive evaluation of how different metrics respond to varying degrees of rotational perturbations.

In the second phase, multidirectional combinations were analyzed. The number of input directions (2 or 3) was used as the independent variable, and correlations with dosimetric indices, including gamma passing rate, were assessed. The analysis focused on direction count rather than specific directional combinations.

All correlation analyses were performed using Pearson's correlation coefficient, with corresponding *p*‐values reported to assess statistical significance.

## 3 RESULTS

This study retrospectively included radiotherapy QA plans from patients treated with the CyberKnife S7 system at the Department of Radiation Oncology, First Medical Center of the Chinese PLA General Hospital. A total of 69 clinically validated SRT treatment plans were selected. All plans were generated using the MultiPlan system (Accuray®, Sunnyvale, USA) and underwent actual dose measurements. In the first stage, multiple dose distribution measurements were performed under simulated discrete rotational error conditions for each patient, yielding 392 two‐dimensional dose images; in the second stage, single dose distribution tests were conducted under non‐discrete rotational error conditions, providing an additional 42 datasets. Following a rigorous quality control screening to exclude abnormal measurements or incomplete data, 364 datasets from the first stage and 41 from the second stage were ultimately retained for feature extraction and subsequent modeling analysis.

### 3.1 Radiomics feature extraction and analysis

**FIGURE 2:**
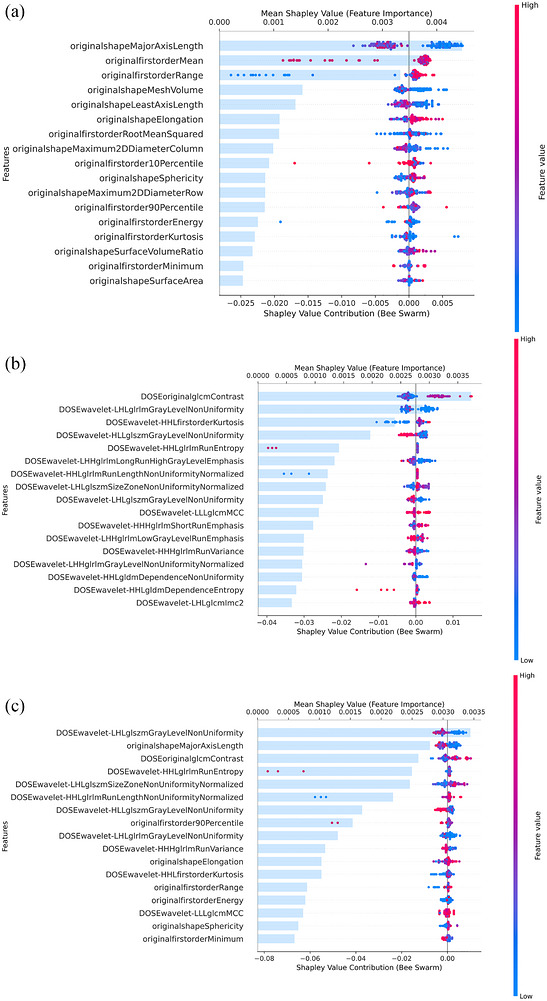
Importance ranking of (A) radiomic features, (B) dosiomic features, and (C) integrated radiomic–dosiomic features.

Radiomics feature extraction was performed on image data resampled to isotropic voxel spacing (1.0 mm × 1.0 mm × 1.0 mm). Intensity discretization was applied with a fixed bin width of 25. According to the study design, only first‐order statistical features and three‐dimensional shape features were extracted, yielding a total of 32 features. (Figure 2(A))

### 3.2 Dosiomic feature extraction and analysis

Dosiomics feature extraction was performed on the processed three‐dimensional dose distribution images. All dose volumes were resampled to match the spatial resolution of the corresponding imaging data, and intensity discretization was applied within the minimum–maximum dose range using a bin width of 1 Gy. On this basis, a total of 837 features were extracted, including first‐order statistical features, texture matrix‐based spatial descriptors (e.g., GLCM, gray‐level dependence matrix [GLDM], GLRLM, and gray‐level size zone matrix [GLSZM]), as well as wavelet‐transformed features (Table 1). As shape features are inherently identical between imaging and dose data, these were not re‐extracted in the dosiomics workflow.

**TABLE 1:** Summary of radiomic and dosiomic features extracted for model development.

Feature Source	Feature Category		Number of Features	
Radiomics	First‐order		18	
	Shape		14	
Dosiomics	First‐order		18	
	Texture	GLCM	24	75
		GLRLM	16	
		GLSZM	16	
		GLDM	14	
		NGTDM	5	
	Wavelet	First‐order	18	744
		GLCM	24	
		GLRLM	16	
		GLSZM	16	
		GLDM	14	
		NGTDM	5	

Feature importance was assessed using an XGBoost model with SHAP. SHAP values quantified the marginal contributions of individual features to the prediction of the SSIM and were used to rank features by importance. Within the radiomics dataset, 17 features were retained, including 9 shape features and 8 first‐order statistical features. Within the dosiomics dataset, 17 key features were identified, consisting primarily of 15 wavelet‐transformed features and 2 texture features. These selected features demonstrated strong explanatory power and significantly contributed to improved model performance. (Figure 2(B)).

### 3.3 Model performance and impact of rotational errors on dose distribution

**FIGURE 3:**
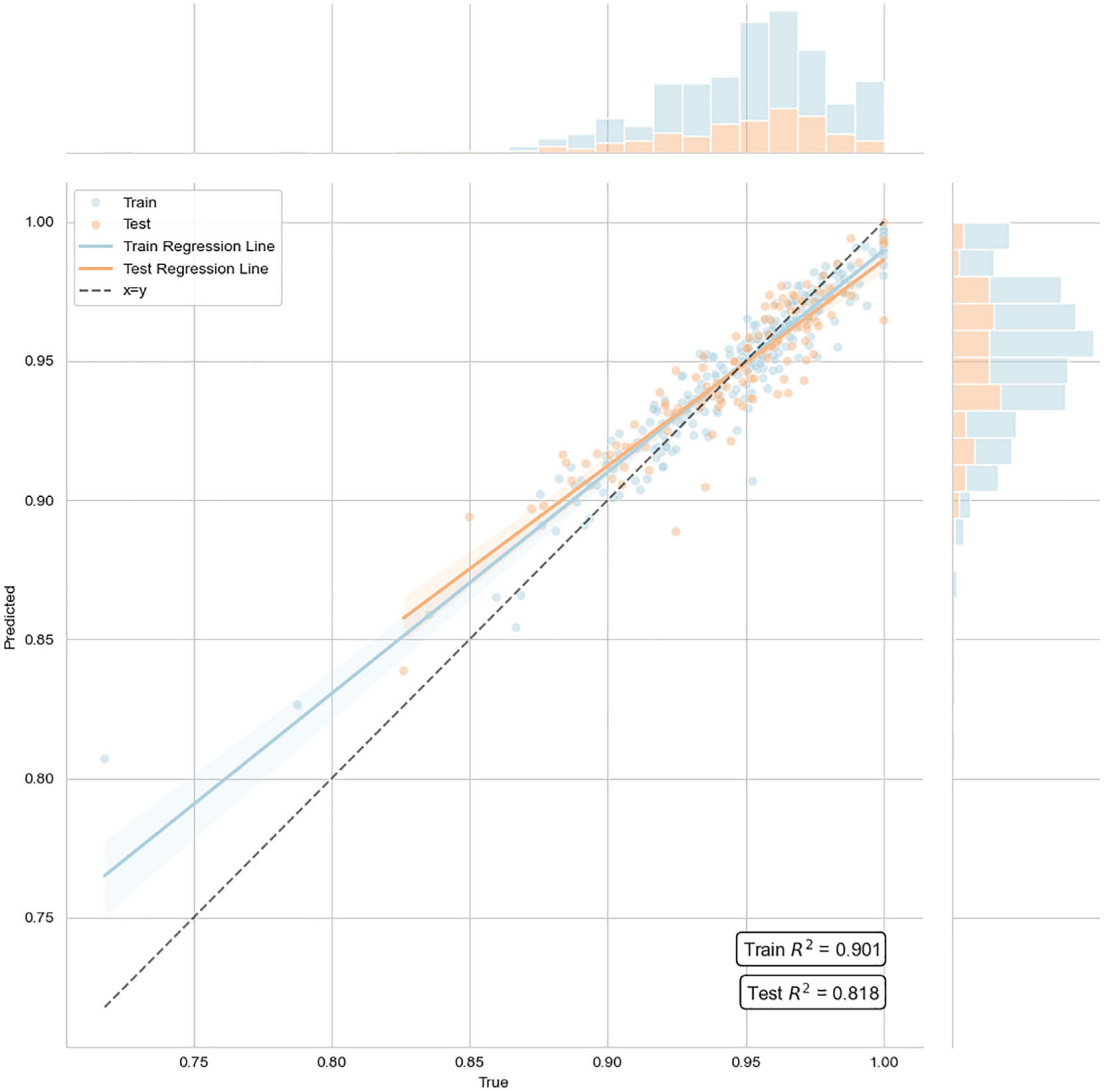
Scatter plot of predicted versus true values for the XGBoost model. Marginal histograms on the top and right display the frequency distributions of the data: coefficient of determination.

**FIGURE 4:**
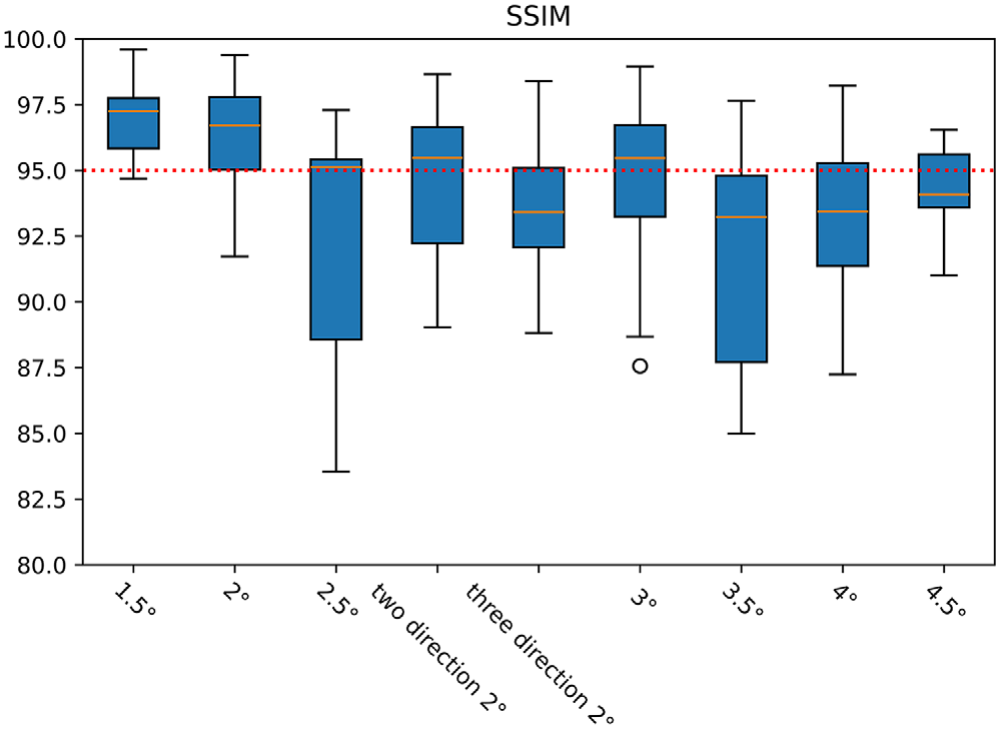
Box plots of SSIM (structural similarity index). SSIM values progressively decrease with increasing rotation angle and directional variation, indicating a decline in dose distribution consistency with accumulated errors.

The overall performance of the model was favorable. The R^2^ reached 0.818, indicating a high degree of goodness‐of‐fit.(Figure 3) More notably, the model demonstrated extremely low error metrics: the mean absolute error (MAE) was approximately 0.0096, the root mean squared error (RMSE) was approximately 0.0123, and the median absolute error (MedAE) was approximately 0.0080. These minimal error values suggest that the deviation between the predicted and actual values was negligible, reflecting a high prediction accuracy. Taken together, the R^2^ and high precision (low errors) indicate that the model achieved reliable performance in predicting SSIM values. (Figure 4)

To further quantify the independent predictive value of radiomic and dosiomic features, we compared the performance of three modeling schemes: (a) a full model incorporating both rotation angles and radiomic/dosiomic features; (b) a model based solely on rotation angles; and (c) a model based solely on radiomic and dosiomic features (Table 3). Among the three models, the full model achieved the best performance (R^2^ = 0.818, MAE = 0.0096, RMSE = 0.0123, and MedAE = 0.0080). The feature‐only model also showed predictive ability (R^2^ = 0.310, MAE = 0.0210, RMSE = 0.0275, and MedAE = 0.0154), outperforming the rotation‐only model (R^2^ = 0.228, MAE = 0.0200, RMSE = 0.0291, and MedAE = 0.0147). These results show that the radiomic/dosiomic feature‐only model outperformed the rotation‐only model, whereas the combined model achieved the highest predictive performance.

This study systematically evaluated the influence of rotational errors on SBRT dose distribution consistency under different directional inputs. Gamma analysis passing rate and SSIM were used as the primary dosimetric endpoints, and Pearson correlation analysis was applied to explore the relationships between dosimetric parameters and angular perturbations.

**FIGURE 5:**
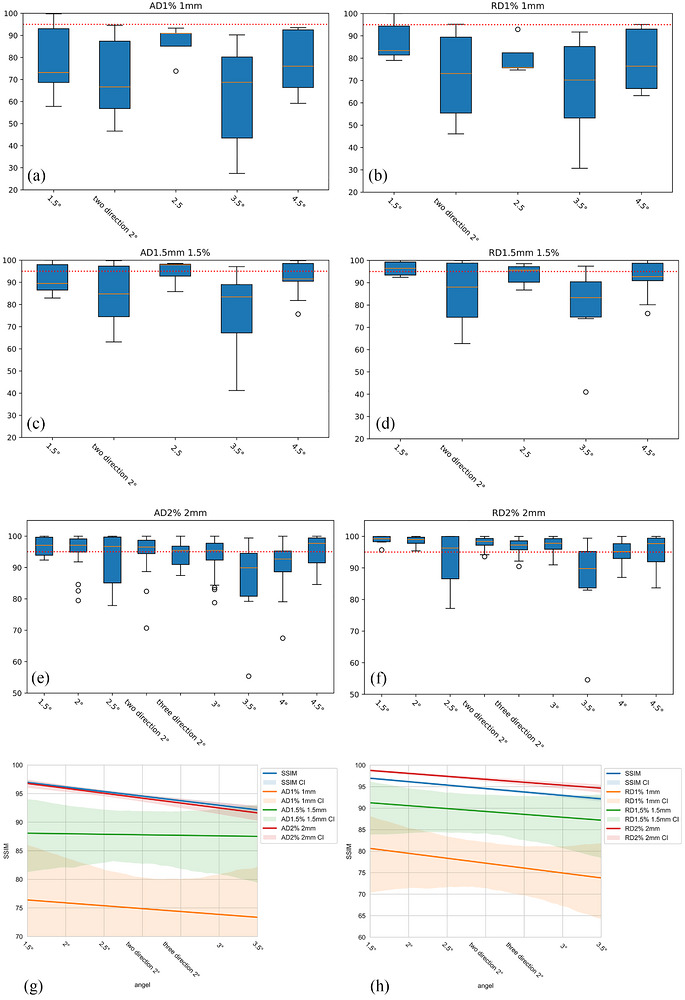
Quantitative evaluation of dosimetric impact under rotational errors using gamma analysis and regression models. (A) Box plots of absolute dose (AD) gamma passing rates with 1%/1 mm criterion. The rates are consistently situated below the 95% clinical acceptance threshold across all rotation angles. (B) Box plots of relative dose (RD) gamma passing rates with 1%/1 mm criterion, showing significant reductions only at large rotational angles. (C) Box plots of absolute dose (AD) gamma passing rates with 1.5%/1.5 mm criterion, which also remain consistently below the 95% clinical threshold. (D) Box plots of relative dose (RD) gamma passing rates with 1.5%/1.5 mm criterion, exhibiting comparable limited sensitivity to rotational perturbations. (E) Box plots of AD gamma passing rates with 2%/2 mm criterion, which also remain consistently below the 95% threshold. (F) Box plots of RD gamma passing rates with 2%/2 mm criterion, exhibiting limited sensitivity with reductions observed primarily at larger errors. (G) Linear regression analysis for Absolute Dose (AD): The solid lines represent the linear fitting of various evaluation metrics against rotational errors under AD conditions. (H) Linear regression analysis for Relative Dose (RD): The solid lines represent the linear fitting of various evaluation metrics under RD conditions, illustrating the correlation trends for each metric.

Under single‐directional inputs, the proportions of plans with gamma passing rates (2%/2 mm) above 95% were 74.7% for CW, 91.6% for RO, and 88.9% for UP (Figure 5(A)). Correlation analysis revealed that SSIM exhibited significant negative correlations with rotation angle across all directions. The strongest correlations were observed in the RO (*r* = −0.747, *p* < 0.001) and UP (*r* = −0.741, *p* < 0.001) directions, while the CW direction (*r* = −0.632, *p* < 0.001) also demonstrated a robust negative association. This consistency confirms SSIM's sensitivity to structural alterations in dose distribution induced by rotational errors (Figure 5(B)).

In contrast to previous observations, the expanded dataset revealed that conventional dosimetric parameters also exhibited statistically significant correlations in most scenarios, though with varying magnitudes. In the CW direction, metrics such as RDDD (*r* = −0.740, *p* < 0.001) and RD2% (*r* = −0.484, *p* < 0.001) showed significant negative correlations. Similarly, in the RO direction, significant correlations were found for RDDD (*r* = −0.666, *p* < 0.001) and RD2% (*r* = −0.577, *p* < 0.001). Notably, even under the intermediate 1.5%/1.5 mm criterion, gamma passing rates failed to show statistical significance in most scenarios (e.g., CW: *p* = 0.880; RO: *p* = 0.349), while SSIM maintained strong correlations (*p* < 0.001). This confirms that SSIM's structural approach captures variations that cannot be simply replicated by tightening gamma thresholds, highlighting the intrinsic insensitivity of the gamma framework to subtle rotational errors compared to SSIM (Figure 5(C)(D), Table 2).

**TABLE 2:** Correlation analysis between rotational errors and dosimetric metrics across different input directions.

Input Direction	Proportion of γ Passing Rate > 95%	Metric	Pearson r	*p*‐value
**CW**	74.7%	RD2% 2 mm	−0.484	< 0.001
		RD1.5% 1.5 mm	−0.064	0.880
		RD1% 1 mm	−0.296	0.477
		RDDD	−0.740	< 0.001
		AD2% 2 mm	−0.445	< 0.001
		AD1.5% 1.5 mm	−0.070	0.868
		AD1% 1 mm	−0.298	0.473
		ADDD2%	−0.580	< 0.001
		**SSIM**	**−0.632**	**< 0.001**
**RO**	74.7%	RD2% 2 mm	−0.577	< 0.001
		RD1.5% 1.5 mm	−0.331	0.349
		RD1% 1 mm	−0.07	0.831
		RDDD	−0.666	< 0.001
		AD2% 2 mm	−0.357	< 0.001
		AD1.5% 1.5 mm	0.146	0.688
		AD1% 1 mm	−0.267	0.457
		ADDD2%	−0.567	< 0.001
		SSIM	−0.747	< 0.001
**UP**	91.6%	RD2% 2 mm	−0.405	< 0.001
		RD1.5% 1.5 mm	0.016	0.812
		RD1% 1 mm	−0.01	0.974
		RDDD	−0.669	< 0.001
		AD2% 2 mm	−0.386	< 0.001
		AD1.5% 1.5 mm	0.086	0.965
		AD1% 1 mm	0.024	0.948
		ADDD2%	−0.567	< 0.001
		SSIM	−0.741	< 0.001
Multi‐directional combination	88.9% / 90.9%	RD2% 2 mm	−0.002	0.742
		RD1.5%1.5 mm	0.147	0.413
		RD1% 1 mm	−0.086	0.645
		RDDD	−0.152	0.01
		AD2% 2 mm	−0.02	0.741
		AD1.5%1.5 mm	0.220	0.219
		AD1% 1 mm	0.285	0.107
		ADDD2%	−0.478	0.211
		SSIM	−0.279	< 0.001

**TABLE 3:** Performance comparison of prediction models with different input features.

Feature Set	R^2^	MAE	RMSE	MedAE
(a) Full Model (Rotation + Features)	0.818	0.0096	0.0123	0.0080
(b) Rotation Angle Only	0.228	0.0200	0.0291	0.0147
(c) Radiomics/Dosiomics Features Only	0.310	0.0210	0.0275	0.0154

For multidirectional inputs, we compared dual‐directional and triple‐directional combinations. The proportions of cases with gamma passing rates > 95% were 88.9% and 90.9%, respectively, with consistently high passing rates, indicating limited sensitivity of gamma analysis to complex directional variability. In correlation analysis, Gamma analysis passing rates lacked statistical significance (*p* > 0.05 for both AD and RD metrics at 2%/2 mm). In contrast, SSIM retained a statistically significant, albeit attenuated, negative correlation with angle (*r* = −0.279, *p* < 0.001). This suggests that while quantification becomes challenging under multi‐orientation data fusion, SSIM maintains superior sensitivity for error detection where conventional metrics fail to demonstrate statistical significance.

### 3.4 Correlation between SSIM and Clinical DVH Metrics

To assess the clinical relevance of SSIM, we performed a preliminary correlation analysis between SSIM and a representative DVH‐based metric. ΔV100, defined as the difference in target V100 between the no‐rotation condition and the rotated condition, was used to quantify the loss of prescription‐dose target coverage induced by rotational error. A statistically significant negative correlation was observed between SSIM and ΔV100 (*r* = −0.625, *p* = 0.004), indicating that lower SSIM values were associated with greater target coverage loss.

## 4 DISCUSSION

This study simulated rotational errors in SBRT to evaluate their dosimetric impact across directions and input combinations. By systematically combining structural‐similarity–based evaluation and quantitative feature analytics, our work bridges the methodological gap between conventional 2D gamma analysis and high‐dimensional data‐driven QA. Dose consistency was assessed with SSIM and conventional metrics, while radiomics/dosiomics modeling explored predictive strategies. This integrative framework not only enhances error detection sensitivity but also establishes a foundation for individualized and automated QA in SBRT, consistent with prior validation studies.^4^, ^10^, ^17^

In the single‐directional analysis, although gamma passing rates varied across directions, SSIM was consistently and significantly negatively correlated with rotation angle (CW: *r* = −0.632; RO: *r* = −0.747; UP: *r* = −0.741; all *p* < 0.001). This consistency across perturbation directions demonstrates that SSIM can capture coherent structural deviations that traditional indices may fail to recognize, particularly in high‐gradient SBRT dose fields where spatial correlations dominate clinical relevance. Gamma‐based QA metrics, while widely adopted, often underestimate small but spatially structured deviations that accumulate into clinically meaningful coverage or OAR‐dose discrepancies.^4^, ^18^, ^19^, ^20^ The inclusion of intermediate gamma criteria (1.5%/1.5 mm) further demonstrates that the lack of sensitivity in conventional metrics is not merely a matter of threshold selection but is intrinsic to the gamma framework's neglect of spatial structural coherence, which SSIM effectively addresses. By contrast, SSIM integrates luminance, contrast, and structural information, yielding a unified, image‐based measure sensitive to geometry‐dependent distortions. Importantly, our exploratory analysis linking SSIM to a representative DVH‐based metric (ΔV100) addresses a common critique of image‐based metrics by demonstrating its clinical relevance. The observed correlation suggests that SSIM is not merely a geometric similarity score but a sensitive predictor of dose‐volume integrity. In practice, a doctor or technologist could use a rapid SSIM assessment to decide whether a full, time‐consuming 3D TPS recalculation is necessary, thereby streamlining the QA workflow without sacrificing clinical safety. This enables more intuitive interpretation of spatial errors by physicists and clinicians and supports direct visualization of dose perturbation patterns.^4^, ^10^ The strongest SSIM–angle correlations were observed in the RO and UP directions, which may reflect plan‐specific beam geometry and anatomy‐dependent weighting, a phenomenon consistent with prior observations that rotational setup errors differentially affect target coverage and nearby critical structures depending on orientation. Collectively, these findings reinforce that SSIM offers higher spatial specificity and structural sensitivity than conventional QA indices, particularly in stereotactic settings with steep dose gradients.^21^, ^22^

To further clarify the clinical implications of SSIM, we emphasize that the value proposed in this study should be interpreted as an exploratory and conservative warning threshold rather than a definitive clinical cutoff. Based on the observed relationship between SSIM and ΔV100, a decrease of approximately 0.03 in SSIM corresponded roughly to a 1% reduction in target V100 in our cohort. Because a reduction in target V100 of more than 2% to 3% would generally prompt additional plan review in SBRT practice, SSIM ≤ 0.95 may serve as a preliminary warning threshold for further verification. This threshold should be understood as a triage reference for additional assessment, not as a direct accept‐or‐reject rule, and it still requires validation across treatment sites, plan complexities, and delivery platforms.

The present study also clarifies the relationship between planar SSIM and clinically relevant three‐dimensional dose information. SSIM was calculated from the two‐dimensional dose plane measured by SRS MapCHECK, whereas DVH metrics require volumetric dose information. Our preliminary analysis showed a statistically significant negative correlation between SSIM and ΔV100 (*r* = −0.625, *p* = 0.004), suggesting that lower SSIM values were associated with greater loss of target prescription‐dose coverage. At the same time, planar measurements cannot fully represent three‐dimensional target and OAR dose‐volume changes. Therefore, SSIM should be interpreted as a rapid, structure‐aware screening indicator that can complement, but not replace, full three‐dimensional DVH and OAR dose evaluation.

The multidirectional input analysis further reinforced these conclusions. While gamma passing rates lacked statistical correlation with rotational errors in multi‐directional scenarios (*p* > 0.05 for both AD and RD metrics), SSIM retained a statistically significant, albeit attenuated, negative correlation (*r* = −0.279, *p* < 0.001). This confirms its superior sensitivity even under complex multi‐orientation fusion where conventional metrics fail to distinguish error magnitudes. Prior studies have similarly noted that gamma pass‐rates may remain high despite meaningful spatial mismatches, limiting error localization and clinical interpretability.^18^, ^19^ SSIM's structural sensitivity helps address this gap by quantifying image‐like deviations in the two‐dimensional (2D) dose domain.^10^

Building upon SSIM analysis, we developed machine‐learning models integrating radiomic and dosiomic features to predict SSIM and identify regions susceptible to rotational perturbations. This approach introduces a predictive layer to QA, transforming similarity evaluation from a retrospective measurement into a prospective, model‐driven quality control tool. The practical advantage of this model‐driven approach is further highlighted by its computational efficiency. While a full 3D dose recalculation for a complex SBRT plan in a clinical TPS typically requires approximately 10 to 15 min per plan, the trained radiomic‐dosiomic model can generate a predicted SSIM value in less than 1 s on a standard workstation. This near‐instantaneous feedback allows for high‐throughput screening of plans, identifying those at risk of significant structural degradation before proceeding with time‐consuming recalculations. Our SHAP‐based analysis identified predominantly wavelet‐derived dosiomic textures among the most influential predictors, consistent with the growing literature showing that dosiomics captures multi‐scale spatial heterogeneity linked to QA outcomes, toxicity, and treatment response.^23^, ^24^ The interpretability achieved via SHAP enhances model transparency and clinical trust,^25^ addressing a key limitation in current AI‐based QA applications. The three‐model comparison further strengthens this interpretation by quantitatively demonstrating that radiomic and dosiomic features provide predictive value independent of rotational error alone. The observation that the feature‐only model outperformed the rotation‐only model suggests that these features capture spatial characteristics of the dose distribution that are not fully represented by geometric error magnitude. Moreover, the markedly superior performance of the full model indicates that rotation angle and radiomic/dosiomic features offer complementary information for predicting SSIM changes. Collectively, these results suggest that the model's predictive ability is not simply driven by the magnitude of the imposed error, but also by its capacity to account for intrinsic spatial properties of the dose field that influence its sensitivity to rotational perturbation. More broadly, emerging work is integrating dosiomics with deep learning for pretreatment prediction of clinical endpoints (e.g., dermatitis, pneumonitis), underscoring the translational potential of feature‐driven dose analytics.^26^, ^27^

Regarding model robustness, the present validation framework was designed to improve internal generalizability, not to establish external validation. By separating treatment plans at the plan level during training and testing, the model was evaluated on previously unseen plan geometries and dose distributions rather than on closely related perturbations from the same plan. This strategy, together with cross‐validation and inclusion of multi‐stage perturbation data, strengthens internal robustness; however, it does not substitute for independent external validation across institutions, platforms, and patient cohorts.

The dominant contribution of wavelet‐derived dosiomic features is also physically plausible. Rotational errors do not simply scale the dose distribution uniformly; rather, they introduce localized structural changes, including displacement of high‐dose edges, distortion of isodose contours, and perturbation of steep dose‐gradient regions. The stronger correlations observed in the RO and UP directions may plausibly reflect stronger coupling between those rotational components, the dominant dose‐gradient geometry, and the detector sampling plane. Wavelet decomposition is well suited to capture such multiscale structural changes because low‐frequency components describe the global dose shape, whereas higher‐frequency components reflect local edges, texture variation, and spatial heterogeneity. The high SHAP importance of wavelet‐based features therefore supports the interpretation that rotational perturbations in SBRT are primarily expressed as spatially structured, multiscale dose alterations.

Our workflow—combining rotational‐error simulation, SSIM assessment, and multimodal feature modeling—demonstrates that structure‐aware metrics and dose‐feature analytics can surpass conventional QA surrogates for plan consistency and error detection.^10^ Rotational errors are hard to correct in real time and can compromise coverage or raise OAR dose; uncorrected rotations ≥ 3° and residual tracking errors in robotic radiosurgery may cause clinically significant deviations.^21^ Incorporating SSIM into QA pipelines could improve structural sensitivity and spatial interpretability, while dosiomics/machine learning (ML) provides a pathway toward individualized, image‐based error prediction and plan adaptation.^23^, ^24^ Parallel advances in data‐driven dose prediction (radiomics/dosiomics with classical ML and deep learning (DL)) further support integrating predictive analytics into planning and QA ecosystems.^28^, ^29^ This study primarily focused on the analysis of rotational errors within the context of SBRT and the CyberKnife system. In clinical practice, however, most conventional radiotherapy equipment lacks the capability for 6D couch adjustment or real‐time rotational correction, making it impossible to compensate for such errors. Therefore, there is a widespread and pressing clinical need to establish an effective evaluation framework capable of accurately quantifying the impact of rotational errors on dose distribution. In this context, the methodology and findings presented in this study can provide valuable insights and references for future research in this direction.

This study has several limitations. Phantom‐based experiments, while well‐controlled, simplify patient setup complexity; literature shows case‐ and site‐dependent sensitivity of dose to rotational errors, cautioning against over‐generalization.^22^, ^30^ To strengthen the validity of our findings, we employed a plan‐level validation strategy to mitigate the risk of data leakage inherent in augmented datasets. By ensuring that the training and test sets contained mutually exclusive treatment plans, we demonstrated that the model's predictive capability extends to new, previously unencountered dose distributions. Our sample size may limit model generalizability; multi‐institutional data and prospective validation are warranted, echoing recommendations from QA predictive‐value assessments.^18^ Finally, although SSIM effectively captures structural changes, its direct linkage to target/OAR dose–volume endpoints and clinical outcomes requires correlation with 3D dose accumulation and outcomes datasets, as suggested by recent dosiomics/toxicity and outcome‐prediction studies.^27^, ^31^

## 5 CONCLUSION

This study demonstrated that the structural similarity index (SSIM) outperforms conventional gamma‐based QA metrics in detecting rotational setup errors in SBRT, offering greater spatial specificity and sensitivity. SSIM showed strong negative correlations with rotation angles across all directions, confirming its robustness to geometry‐dependent distortions. Combined with radiomic and dosiomic modeling, SSIM enabled predictive, data‐driven QA. These findings highlight the potential of SSIM‐based, structure‐aware frameworks to improve interpretability and automation in clinical QA, warranting further validation with multi‐institutional and outcome‐linked datasets. Moreover, the observed correlation between SSIM and a representative clinical DVH‐based metric (ΔV100) supports its potential utility as a clinically grounded screening tool for SBRT dose perturbations.

## AUTHOR CONTRIBUTIONS

Conceptualization, Rui Qi and Jinglin Sun; Methodology, Rui Qi and Jinglin Sun; Software, Rui Qi; Formal analysis, Rui Qi and Tingyu Mu; Investigation, Hongji Shi and Jiayi Zhang; Resources, Jinglin Sun; Writing—original draft, Rui Qi and Tingyu Mu; Writing—review and editing, Rui Qi and Xiangkun Dai; Visualization, Rui Qi and Tingyu Mu; Supervision, Xiangkun Dai; Funding acquisition, Xiangkun Dai. All authors have read and agreed to the published version of the manuscript.

## CONFLICT OF INTEREST STATEMENT

The authors declare no conflicts of interest.

## ETHICS STATEMENT

This study was approved by the Institutional Review Board of the First Medical Center of Chinese PLA General Hospital (Approval No. S2025‐1124‐01). The requirement for informed consent was waived due to the retrospective design and the use of de‐identified data.

## Supporting information



Supporting Information

## Data Availability

The data that support the findings of this study are available from the corresponding author upon reasonable request.
