# Supplementary material for: Assessment of rotational dose perturbations in SBRT: A radiomic‐dosiomic predictive model based on the structural similarity index measure
Source: J Appl Clin Med Phys. 2026 May 26;27(5):e70634. doi: 10.1002/acm2.70634 (PMC13239961; doi:10.1002/acm2.70634)
Supplement: Supplementary file 1 — Supporting Information [file ACM2-27-e70634-s001.docx]

Table S1. Detailed distribution of rotational error scenarios and sample sizes.

| Scenario ID | Rotational Vector (RO, UP, CW​)/∘ | Sample Size (n) | Error Dimensionality |
| --- | --- | --- | --- |
| 1-3 | (2,0,0),(3,0,0),(4,0,0) | 28 | Single-Axis |
| 4-6 | (0,2,0),(0,3,0),(0,4,0) | 28 | Single-Axis |
| 7-9 | (0,0,2),(0,0,3),(0,0,4) | 28 | Single-Axis |
| 10-13 | (1.5,0,0),(2.5,0,0),(3.5,0,0),(4.5,0,0) | 3 | Single-Axis |
| 14-17 | (0,1.5,0),(0,2.5,0),(0,3.5,0),(0,4.5,0) | 3 | Single-Axis |
| 18-21 | (0,0,1.5),(0,0,2.5),(0,0,3.5),(0,0,4.5) | 3 | Single-Axis |
| 22 | (2,2,0) | 30 | Multi-Axis (Dual) |
| 23 | (0,2,2) | 30 | Multi-Axis (Dual) |
| 24 | (2,0,2) | 30 | Multi-Axis (Dual) |
| 25 | (2,2,2) | 28 | Multi-Axis (Triple) |
